# Dietary supplementation with Chinese herbal mixture extracts enhances growth performance, immunity, antioxidant capacity, and intestinal microbiota function in calves

**DOI:** 10.3389/fvets.2025.1530124

**Published:** 2025-04-08

**Authors:** Mingxi Zhang, Di Shen, Yongxing Wu, Donghe Dang, Shuwei Dong, Jingyan Zhang

**Affiliations:** ^1^College of Life Sciences, Yulin University, Yulin, Shanxi, China; ^2^Lanzhou Institute of Husbandry and Pharmaceutical Sciences, Chinese Academy of Agricultural Sciences, Lanzhou, Gansu, China; ^3^Gansu Qingliang Yuan Biological Medicine Co., Ltd., Dingxi, Gansu, China; ^4^Xian Caotan Animal Husbandry Co., Ltd., Xian, Shanxi, China; ^5^Cell Biology and Immunology Group, Wageningen University & Research, Wageningen, Netherlands

**Keywords:** Chinese herbal mixture extracts, growth performance, immunity, antioxidant capacity, intestinal microbiota, calves

## Abstract

This study examined the effects of dietary supplementation with Chinese herbal mixture extracts (CHE) on growth performance, Immunity, antioxidant capacity, and gut microbiota composition in dairy calves. CHE is a compound extracts powder composed of *Honeysuckle, Astragalus, Officinal magnolia bark*, and *Tangerine peel*. Forty calves were randomly assigned to four groups: basal diet (CON), basal diet + 0.1% CHE (LCHE), basal diet + 0.2% CHE (MCHE), and basal diet + 0.4% CHE (HCHE). The experiment was conducted for 56 days with daily observations, bi-weekly weighing, blood sampling, and fecal collection toward the end. The addition of the CHE group significantly increased the average daily weight gain (ADG) and decreased the feed/gain ratio (F/G) compared to the CON group (*p* < *0.05*). The apparent digestibility of crude fat, neutral detergent fiber, and acid detergent fiber was higher in HCHE and MCHE groups (*p* < *0.05*). Serum GH and IGF-1 levels increased in MCHE and HCHE groups (*p* < *0.05*). The blood biochemical analysis revealed that the levels of CA and GLU in the MCHE group were higher than those in the CON group, while remaining within the normal physiological range. Both the IgG and IFN-γ levels and the serum antioxidant levels were significantly increased in the CHE supplementation group compared with the control group (*p* < *0.05*). High-throughput 16S rRNA sequencing revealed changes in gut microbiota, with increased *unclassified Muribaculaceae* and *UCG-005* species in MCHE and HCHE groups (*p* < *0.05*). In conclusion, CHE supplementation enhanced digestion, growth performance, immunity, and gut microbiota balance in calves without toxic side effects.Considering both the economic benefits and the effects of the additive, a clinical dosage of 0.2% CHE additive may be recommended.

## 1 Introduction

The digestive and metabolic systems of newborn calves are not yet fully developed, resulting in significantly inadequate digestive and metabolic performance and a limited ability to digest solid food. Therefore, calves at this stage are mainly fed whole milk or milk replacer (1), which significantly increases feeding costs. Calves at this stage are very sensitive to environmental changes and pathogenic bacteria, making them susceptible to diseases such as diarrhea and pneumonia, which can severely hinder normal growth and development may even lead to death, posing numerous challenges for farm management (2). Currently, In-feed antibiotics have been widely applied to nursery diets for controlling post-weaning diarrhea and promoting animal health and growth (3). However, in large-scale intensive livestock systems, the inappropriate use of antibiotics can disrupt the gut microbiota, impair immunity, promote antibiotic resistance, and lead to residues that pose risks to human health and the environment (4, 5).

Compared with chemical medicines, traditional Chinese herbs have certain advantages in the prevention and treatment of animal diseases, such as lower toxicity, no resistance, and multiple effects including immune modulation, anti-oxidation, and health maintenance, which makes them important agents for the preventive and treatment of calf diseases (6, 7). Herbal additives based on traditional Chinese herbal theory and produced from various herbs through special processes are healthy and environmentally friendly feed additives. After being fully absorbed by the animal body, their nutrients can regulate physiological functions, promote nutrient absorption, improve health status, and enhance the quality of animal products (8). Moreover, herbal additives are rich in organic compounds such as alkaloids, polysaccharides, and saponins, which can effectively improve metabolic functions (9, 10). Chinese herbal supplementation during the perinatal period in dairy cows has been shown to enhance reproductive performance, elevate antioxidant capacity, and strengthen neonatal calf immunity (11). Dietary inclusion of compound herbal formulations in weaned calves significantly improves growth parameters and modulates immune responses (12). Similarly, dietary supplementation with herbal extracts in Holstein calves has been found to enhance immune function and antioxidant activity, thereby effectively promoting overall health (13). Furthermore, the incorporation of blended herbal mixtures into calf rations reduces physiological oxidative stress and increases antioxidant levels (14).

*Honeysuckle, Astragalus, Officinal magnolia bark*, and *Tangerine peel* are medicinal and edible Chinese herbal medicines widely used in China since ancient times. These Chinese herbal medicines not only contain carbohydrates, proteins, crude fats, crude fibers, vitamins, minerals and other nutrients but also organic acids, flavonoids, volatile oils, triterpenoid saponins and other active ingredients, which can be used as feed additives in livestock and poultry production (15). *Honeysuckle* is an important medicine for clearing heat and detoxifying agent that has anti-inflammatory, antibacterial, antiviral, anti-tumor hypoglycemic, antioxidant, immunity-boosting and other beneficial effects (16). Studies have shown that *Honeysuckle* extracts, particularly those with varying chlorogenic acid content, can enhance the production performance of Holstein cows, improve rumen fermentation conditions, and boost antioxidant capacity and immune function (17). *Astragalus*, known for its qi-tonifying and immune-regulating properties, exhibits a wide range of biological activities, including cardiovascular and cerebrovascular protection, anti-tumor effects, anti-inflammatory responses, anti-aging properties, and antioxidant activity (18). Dietary supplementation with *Astragalus*-based preparations in broiler feed improves feed efficiency and enhances antioxidant capacity (19). *Officinal magnolia bark*, traditionally used for drying dampness and resolving phlegm, has been found to regulate gastrointestinal hormones, modulate material metabolism, protect the intestinal barrier, and influence gut microbiota composition, thereby contributing to disease prevention and treatment (20). *Tangerine peel*, recognized for its qi-regulating and spleen-strengthening effects, has been demonstrated to improve digestive and absorptive functions, enhance antioxidant activity, and exert antibacterial and bacteriostatic effects. Dietary supplementation with Tangerine peel in broiler feed improves growth performance, immune function, and antioxidant status (21). Based on these findings, we selected *Honeysuckle, Astragalus, Officinal magnolia bark*, and *Tangerine peel* to formulate a Chinese herbal mixture according to the principles of traditional Chinese medicine, and processed into the extracts. But the effects of this Chinese herbal mixture extracts as feeding additives in calves was unknown. Therefore, this study aimed to determine the efficacy and optimal dosage of the extracts in calves by analyzing the change on the growth performance, immune function, and antioxidant capacity at three doses. The research findings can provide scientific evidence to support the application of traditional Chinese herbal extract as feed additives for calves.

## 2 Materials and methods

### 2.1 The CHE preparation

The Chinese herbal mixture extracts (CHE) was composed of *Honeysuckle, Astragalus, Officinal magnolia bark, Tangerine peel*, and their ratio was 1:1:1:1. After cutting, drying, and mixing, the chinese herbal mixture we extracted and concentrated with 10 volumes of distilled water by boiling, the Chinese herbal mixture extracts with a concentration of more than 1.05 g · mL ^−1^ was produced. Subsequently, the extracts were spray-dried to obtain the powder, which was characterized as a yellow-brown powder with a subtle aromatic scent. The extract powder exhibited solubility in both water and milk and demonstrated good palatability.

### 2.2 Animal treatment and experimental design

The experiment was conducted in a large dairy farm in Xi'an, Shaanxi Province. Forty healthy Holstein calves, about 10 days old and with similar body weight (41.91 ± 4.37 kg), were randomly divided into four groups: including three groups with different dosages of CHE (LCHE: basal diet + 0.1% CHE; MCHE: basal diet + 0.2% CHE; HCHE: basal diet + 0.4% CHE) and one control (CON: basal diet) group, with 10 calves in each group. The CHE was mixed with milk at different doses, while the CON group was fed only milk and starter feed. The pre-test period was 3 days and the formal test period was 56 days. The calves were fed whole milk and starter feed in accordance with the farm's production practices. The whole milk was prepared by mixing milk replacer powder with warm water, and the starter feed consisted mainly of corn (50.0%), soybean meal (37.5%), cottonseed meal (10.0%), limestone (1%), sodium chloride (0.5%), vitamins and trace elements (1%). The nutritional components of the milk replacer and the starter feed are shown in Table 1. The required milk replacer powder and starter feed were provided by the dairy farm. The pelleted premix was supplemented based on actual intake, without restriction, and whole milk was fed daily at 06:30, 13:30 and 20:00 h. Each calf was housed individually to prevent cross-infection. All feeding and management conditions were kept consistent across all groups during the experimental period.

**Table 1:** Nutrient levels of forages.

**Items**	**Milk substitute powder (%)**	**Feed (%)**
Water content	≤ 5.2	≤ 14.0
CP	≥22	≥20.0
Ash	≤ 10.0	≤ 10.0
CF	≤ 0.3	≤ 12.0
NaCl	0.6–1.4	0.05–2.0
AP	≥0.6	≥0.3
CA	0.6–1.2	0.4–1.7
Lysine	≥1.7	≥0.3
EE	≥16.05	—
Lactose	≥35.0	—

### 2.3 Sample collection

On days 0, 14, 28, 42 and 56 of the experiment, 10 ml of blood was collected from the jugular vein of each calf using a vacuum collection vessel without any additives. The collected whole blood samples were stored at 4°C for 12 h, then centrifuged at 4,000 rpm for 10 min, and the serum was collected and stored at −20°C for subsequent analysis.

Three days before the end of the experiment, 200 g of feces were randomly collected daily from each calf. Thirty percent of the fecal sample was mixed with 10 % sulfuric acid (0.2 mL/g) for nitrogen fixation and stored at −20°C for crude protein determination. Seventy percent of the fecal samples were stored in sealed bags at −20°C and labeled for determination of the apparent digestibility of nutrients in the feces. At the end of the experiment, 1 to 2 g of feces was collected in a 1.5 mL Eppendorf tube using an anal swab and rapidly stored at −20°C for subsequent fecal microbiota analysis.

### 2.4 Determination of growth performance indicators

Before feeding, the weight of the amount remaining from the previous meal and the amount added were recorded and used to calculate the average daily feed intake (ADFI). The ADFI of calves refers to the sum of daily dry matter content in the milk and the starter feed.


$$\begin{array}{l} \text{ADFI}=\left(\text{daily feeding}-\text{daily surplus material}\right)/\text{test days} \end{array}$$

The experimental cattle were weighed before midday feeding on days 0, 14, 28, 42, and 56, recorded in head units and the average daily weight gain (ADG) and feed-to-gain ratio (F/G) were calculated.


$$\begin{array}{l} \text{ADG}=\left(\text{final weight}-\text{initial weight}\right)/\text{test days}\\ \text{F}/\text{G}=\text{feed volume consumed during the test period}/\text{weight}\\ \text{ gain in the same period} \end{array}$$

### 2.5 Determination of apparent digestibility of nutrients

The dry matter (DM), crude protein (CP), crude fat (EE), acid detergent fiber (ADF), and neutral detergent fiber (NDF) in feed and feces were determined. The determination steps refer to the “Feed Analysis and Feed Quality Detection Technology” and the determination was based on the following standards: water content: GB/T 20195-2006, GB/T 6435-2014 (22); crude protein: GB/T 6432-2018 (23); crude fat: GB/T 6433-2006 (24); neutral detergent fiber: GB/T 20806-2022 (25); acid detergent fiber: NY/T 1459-2022 (26).

Apparent digestibility of nutrients in feed (%) = [mass of nutrients ingested (g) – corresponding mass of nutrients in feces (g)]/mass of nutrients ingested (g) × 100%.

### 2.6 Serum indicators

#### 2.6.1 Growth hormone in serum

The content of growth hormone (GH) and insulin-like growth factor-1 (IGF-1) in serum on days 0, 28, and 56 were determined using a one-step sandwich enzyme-linked immunosorbent assay (ELISA) kit from Shanghai Enzyme-linked Biotechnology Co., Ltd. (GH: ml610251; IGF-1: ml002483) The test was performed strictly according to the instructions of the kit.

#### 2.6.2 Serum biochemical indicators

The contents of total protein (TP), alanine aminotransferase (ALT), aspartate aminotransferase (AST), creatinine (Cr), blood urea nitrogen (UREA), glucose (GLU), and calcium (Ca) in serum were measured using biochemical detection kits from Meikang Biotechnology Co., Ltd. (TP: MJ102; ALT: MJ001; AST: MJ002; Cr: MJ105; BUN: MJ106W; GLU: MJ109; Ca: MJ303). The tests were performed strictly according to the instructions of the kit.

#### 2.6.3 Serum immunoglobulin and inflammatory factors

The serum levels of immunoglobulins IgA, IgG, and inflammatory factors IL-8 and IFN-γ were measured on days 0, 14, 28, 42, and 56 of the experiment using ELISA kits produced by Jiubang Biological Technology Co., Ltd. (IgA: QZ-11414; IgG: QZ-11416; IL-8: QZ-11413; IFN-γ: QZ-16216). These indicators were analyzed by Jiubang Biological Technology Co., Ltd.

#### 2.6.4 Serum antioxidant indices

The total antioxidant capacity (T-AOC), superoxide dismutase (SOD), malondialdehyde (MDA), and glutathione peroxidase (GSH-PX) in serum were measured on days 0, 14, 28, 42, and 56 of the experiment using antioxidant-related kits produced by Nanjing Jiancheng Bioengineering Research Institute (T-AOC: A015-2-1; SOD: A001-3; MDA: A003-1; GSH-PX: A005-1). The experiments were performed strictly according to the instructions of the kit.

### 2.7 Analysis of fecal microbiota

The genomic DNA of the feces was extracted using a Stool DNA Kit (Tiangen Biotech (Beijing) Co., Ltd.) according to the manufacturer's instructions (DP705). The V1-V9 hypervariable regions of the 16S rRNA gene were amplified using primers (27F: AGRGTTTGATYNTGGCTCAG; 1492R: TASGGHTACCTTGTTASGACTT). The amplicons were quantified, and normalized equimolar concentrations of the amplicons were pooled and sequenced on the PacBio Sequel II platform (Beijing Biomarker Technologies Co., Ltd., Beijing, China).

The bioinformatics analysis of this study was performed with the aid of the BMKCloud (http://www.biocloud.net/). The raw reads generated from sequencing were filtered and demultiplexed using the SMRT Link software (version 8.0) with the minPasses ≥5 and minPredictedAccuracy ≥0.9, in order to obtain the circular consensus sequencing (CCS) reads. Subsequently, the Lima (version 1.7.0) was employed to assign the CCS sequences to the corresponding samples based on their barcodes. CCS reads containing no primers and those reads beyond the length range (1,200–1,650 bp) were discarded through the recognition of forward and reverse primers and quality filtering using the Cutadapt (version 2.7) quality control process. The UCHIME algorithm (v8.1) was used in detecting and removing chimera sequences to obtain clean reads. Sequences with similarity >97% were clustered into the same operational taxonomic unit (OTU) by USEARCH (v10.0), and the OTUs counts <2 in all samples were filtered.

Clean reads then were conducted on feature classification to output ASVs (amplicon sequence variants) by DADA2, and the ASVs counts <2 in all samples were filtered. Taxonomy annotation of the OTUs/ASVs was performed based on the Naive Bayes classifier in QIIME2 using the SILVA database (release 138.1) with a confidence threshold of 70%. The Alpha diversity was calculated and displayed by the QIIME2 and R software, respectively. Beta diversity was determined to evaluate the degree of similarity of microbial communities from different samples using QIIME. Principal coordinate analysis (PCoA), heatmaps, UPGMA and nonmetric multidimensional scaling (NMDS) were used to analyze the beta diversity. Furthermore, we employed Linear Discriminant Analysis (LDA) effect size (LEfSe) to test the significant taxonomic difference among groups. A logarithmic LDA score of 4.0 was set as the threshold for discriminative features. To explore the dissimilarities of the microbiome among different factors, a redundancy analysis (RDA) was performed in R using the package vegan.

The qualified sequences with more than 97% similarity thresholds were allocated to one operational taxonomic unit (OTU) using USEARCH (version 10.0). Taxonomy annotation of the OTUs/ASVs was performed based on the Naive Bayes classifier in QIIME2 using the SILVA database (release 138.1) with a confidence threshold of 70%. Alpha was performed to identify the complexity of species diversity of each sample utilizing QIIME2 software. Beta diversity calculations were analyzed by principal coordinate analysis (PCoA) to assess the diversity in samples for species complexity. One-way analysis of variance was used to compare bacterial abundance and diversity. Linear discriminant analysis (LDA) coupled with effect size (LEfSe) was applied to evaluate the differentially abundant taxa. The online platform BMKCloud (https://www.biocloud.net) was used to analyze the sequencing data.

### 2.8 Statistical analysis

The experimental data were analyzed for significance of differences by One-WayANOVA using SPSS27.0 statistical software, and Duncan's method was used for multiple comparisons and tests, with *p* < 0.05 indicating a significant difference, and *p* > 0.05 indicating a non-significant difference. Correlation analysis between intestinal flora and growth performance was performed with Spearman and graphing was performed with GraphPadPrism software.

## 3 Results

### 3.1 Growth performance

#### 3.1.1 Effects on feed intake and body weight

**Figure 1:**
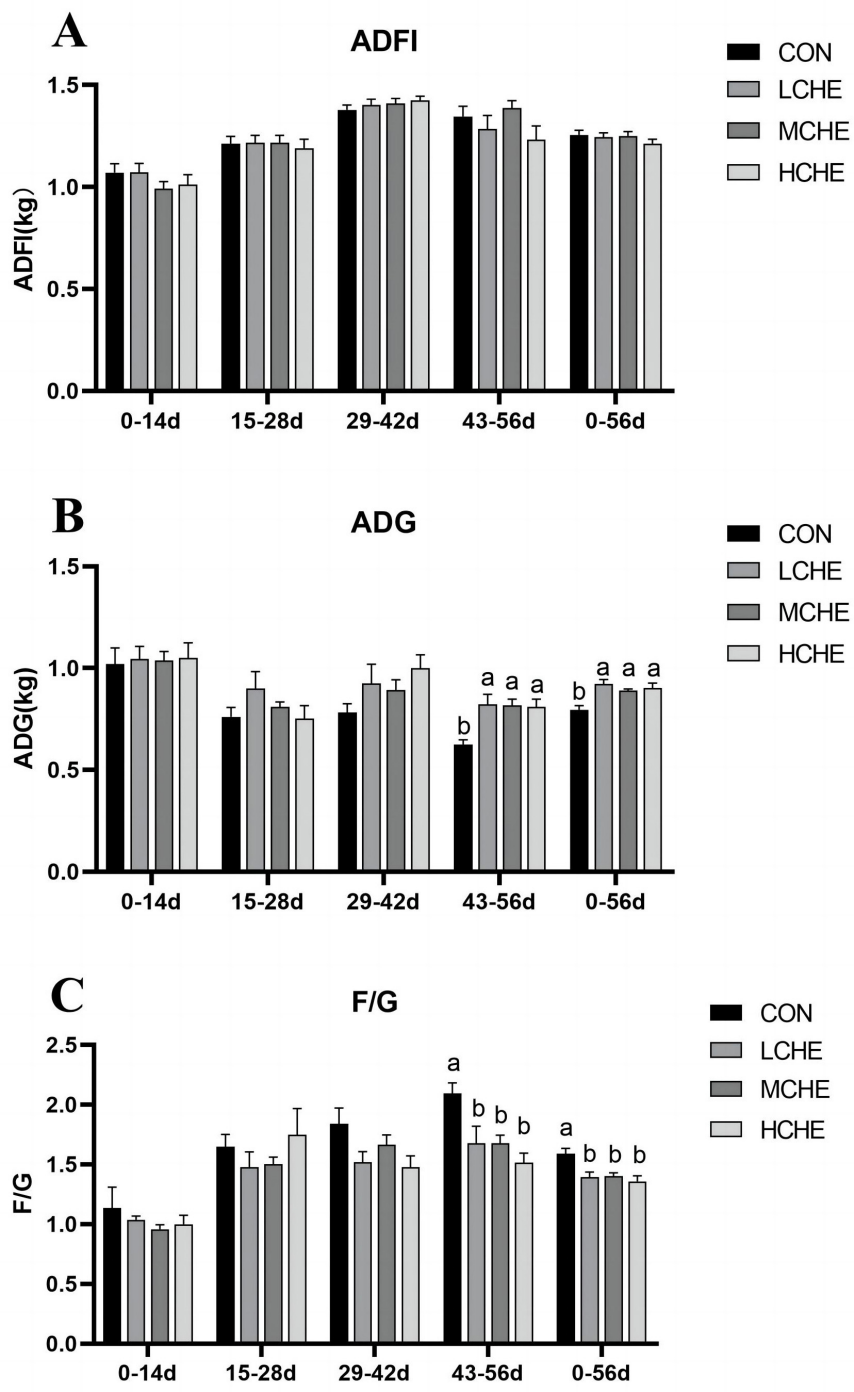
Effects of CHE on feed intake and body weight of calves. **(A)** calves average daily feed intake level. **(B)** calves average daily weight level. **(C)** the feed-to-weight ratios level. CON group (fed basal diet), LCHE (basal diet + 0.1% CHE), MCHE (basal diet + 0.2% CHE), HCHE (basal diet + 0.4% CHE). Mean ± SEM are shown (*n* = 8).Significant differences (*p* < *0.05*) are denoted by the distinct letters a and b.

As shown in on Figure 1, there was no significant difference in average daily feed intake (ADFI) between the CHE groups and the CON group (*p* > *0.05*) (Figure 1A). From day 0 to day 56 and from day 43 to day 56, the average daily weight gain of all CHE groups was significantly higher than that of the CON group (*p* < *0.05*) (Figure 1B). During these periods, the feed-to-weight ratios were significantly lower in the CHE groups than in the CON group (*p* < *0.05*). Additionally, there was no significant difference among the high, medium, and low dosage groups (*p* > *0.05*) (Figure 1C).

#### 3.1.2 Effect on apparent digestibility of nutrients

**Figure 2:**
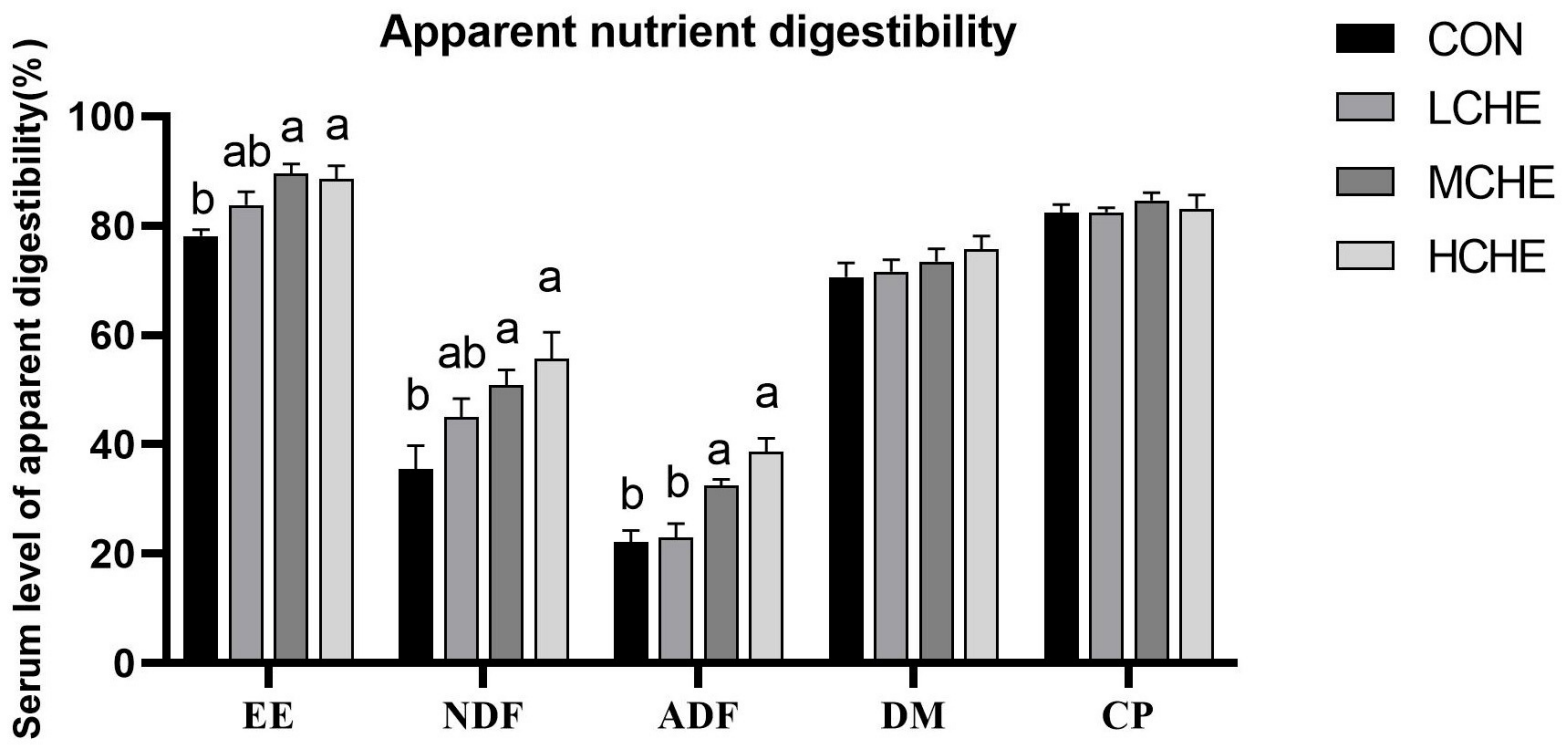
Effects of CHE on Apparent nutrient digestibility of calves. EE, crude fat. NDF, neutral detergent fiber;ADF, acid detergent fiber; DM, dry matter; CP, crude protein. CON group (fed basal diet), LCHE (basal diet + 0.1% CHE), MCHE (basal diet + 0.2% CHE), HCHE (basal diet + 0.4% CHE). Mean ± SEM are shown (*n* = 5).Significant differences (*p* < *0.05*) are denoted by the distinct letters a and b.

Figure 2 shows that there was no significant difference in the apparent digestibility of dry matter and crude protein between the CHE groups and the CON group (*p* > *0.05*). However, the apparent digestibility of crude fat, neutral and acid detergent fibers was significantly higher in the HCHE and MCHE groups than in the CON group (*p* < *0.05*). The MCHE group showed the best digestibility of crude fat, while the HCHE group showed the best digestibility for neutral and acid detergent fibers.

#### 3.1.3 Effect on serum growth hormone

**Figure 3:**
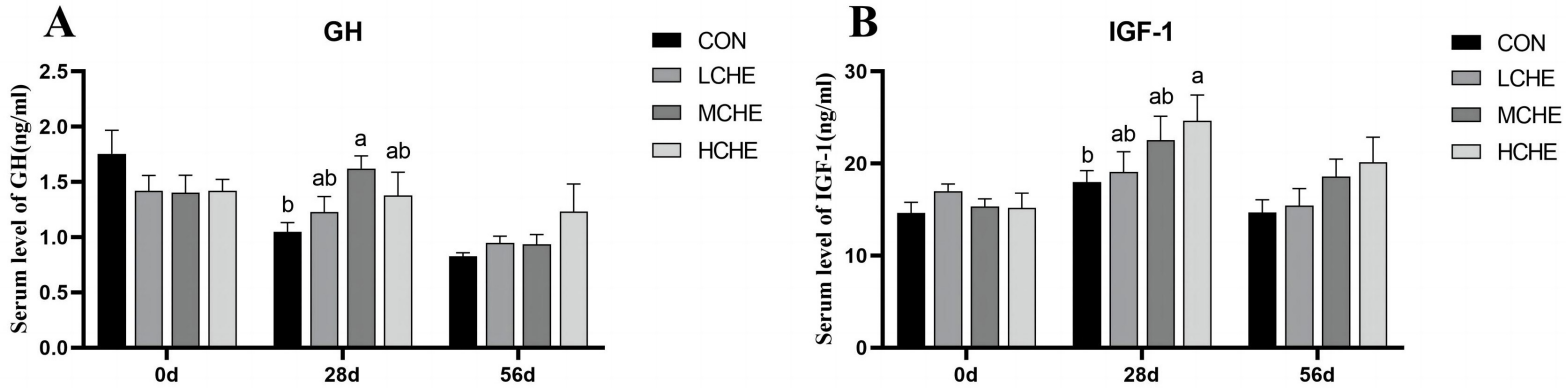
Effects of CHE on serum growth hormone of calves. **(A)** Serum GH level. **(B)** Serum IGF-1 level. CON group (fed basal diet), LCHE (basal diet + 0.1% CHE), MCHE (basal diet + 0.2% CHE), HCHE (basal diet + 0.4% CHE). Mean ± SEM are shown (*n* = 8). Significant differences (*p* < *0.05*) are denoted by the distinct letters a and b.

Figure 3 shows that the GH content in the serum of the MCHE group was significantly higher than that of the CON group on day 28 of the experiment (*p* < *0.05*) (Figure 3A). The serum IGF-1 level of the HCHE group was significantly higher than that of the CON group (*p* < *0.05*), while the low- and MCHE groups showed higher IGF-1 levels compared to the CON group, but the differences were not significant (*p* > *0.05*) (Figure 3B). In addition, there were no significant differences between the high, medium and LCHE groups (*p* > *0.05*).

### 3.2 Serum biochemical indicators

Table 2 shows that there were no significant differences between the CHE groups in the biochemical indicators in the blood on day 0 of the trial (*p* > *0.05*). On trial day 28, the serum Ca content was significantly higher in the MCHE and HCHE groups than in the CON group and the LCHE group (*p* < *0.05*). On day 56 of the trial, the serum GLU level was higher in the CHE groups than in the CON group, with the LCHE and MCHE groups being significantly higher than the CON group (*p* < *0.05*). There were no significant differences among the CHE groups (*p* > *0.05*).

**Table 2:** Results of blood biochemical indicators (*n* = 8).

**Items**	**Treatments**				**SEM**	***p*-value**
	**CON**	**LCHE**	**MCHE**	**HCHE**		
**0 days of age**						
TP (g/L)	69.15	66.58	67.07	66.33	2.05	0.967
CREA (μmol/L)	38.88	43.20	41.45	43.98	1.26	0.517
GLU (mmol/L)	2.95	2.47	2.72	2.15	0.18	0.473
CA (mmol/L)	2.47	2.15	2.52	2.28	0.08	0.345
UREA (mmol/L)	3.07	3.18	3.08	3.37	0.15	0.904
ALT (U/L)	7.62	9.68	5.72	5.63	1.15	0.585
**28 days of age**						
TP (g/L)	57.08	55.23	60.75	61.70	1.06	0.091
CREA (μmol/L)	37.25	37.92	40.80	35.57	1.08	0.399
GLU (mmol/L)	2.87	3.08	3.60	2.95	0.15	0.302
CA (mmol/L)	2.07^b^	2.10^b^	2.33^a^	2.33^a^	0.04	0.012
UREA (mmol/L)	2.03	2.13	2.05	2.42	0.08	0.368
ALT (U/L)	7.27	6.40	4.92	5.63	0.62	0.598
**56 days of age**						
TP (g/L)	68.00	63.18	64.47	64.07	1.36	0.638
CREA (μmol/L)	101.03	107.25	97.20	100.32	3.12	0.741
GLU (mmol/L)	2.05^b^	2.70^a^	2.93^a^	2.45^ab^	0.11	0.025
CA (mmol/L)	2.07	2.18	2.12	2.10	0.02	0.384
UREA (mmol/L)	3.20	3.48	3.27	3.53	0.16	0.877
ALT (U/L)	5.65	8.37	12.23	5.58	1.18	0.146

Results of blood biochemical indicators. CON group (fed basal diet), LCHE (basal diet + 0.1% CHE), MCHE (basal diet + 0.2% CHE), HCHE (basal diet + 0.4% CHE). The means are shown (*n*= 8). Within the same row, data with different superscript letters indicate significant differences (*p* < 0.05); data with the same superscript letters or without any letters indicate no significant differences (*p*> 0.05).

### 3.3 Immunoglobulin and inflammatory factors

**Figure 4:**
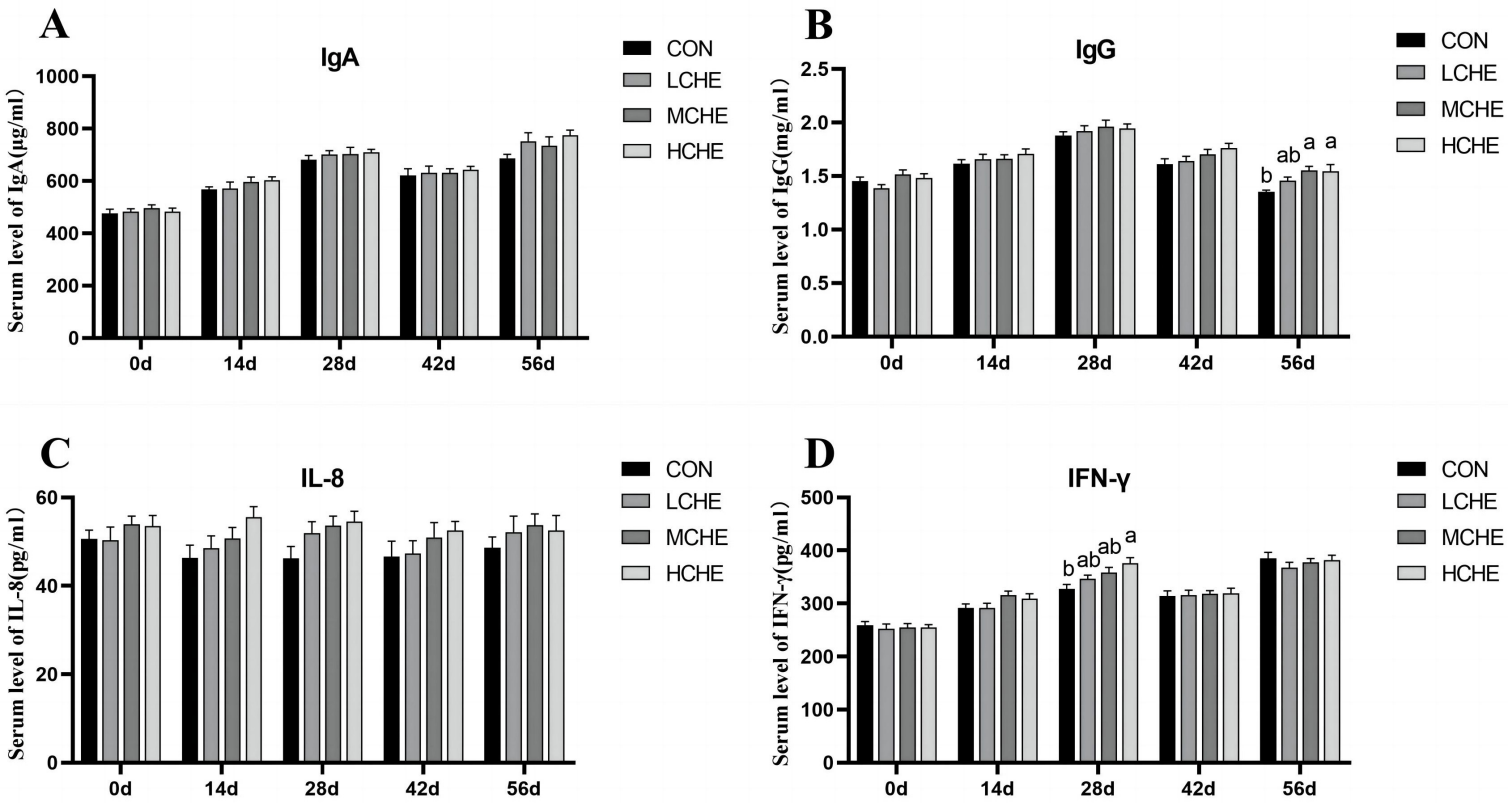
Effects of CHE on serum immunoglobulin of calves. **(A)** Serum IgA level. **(B)** Serum IgG level. **(C)** Serum IL-8 level. **(D)** Serum IFN-γ level. CON group (fed basal diet), LCHE (basal diet + 0.1% CHE), MCHE (basal diet + 0.2% CHE), HCHE (basal diet + 0.4% CHE). Mean ± SEM are shown (*n*= 8).Significant differences (*p* < *0.05*) are denoted by the distinct letters a and b.

Figure 4 shows that on day 28, the IFN-γ content in the serum of the HCHE group was significantly higher than that of the CON group. On day 56, the serum IgG level was significantly higher in the HCHE and MCHE groups than in the CON group (*p* < *0.05*), while the MCHE group was higher than the blank CON group but without a significant difference (*p* > *0.05*). Furthermore, there were no significant differences between the high, medium and LCHE groups (*p* > *0.05*).

### 3.4 Antioxidant index

**Figure 5:**
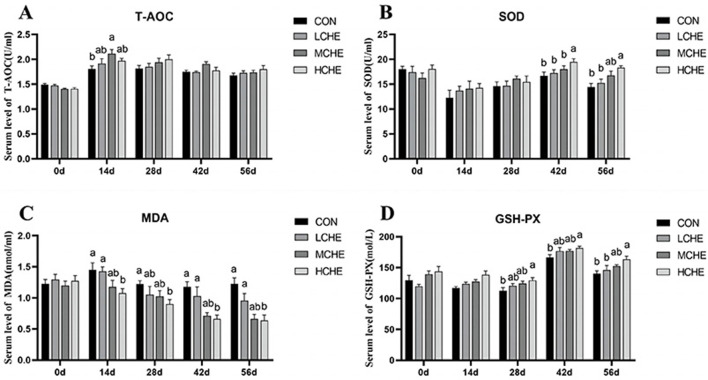
Effects of CHE on serum antioxidant levels of calves. **(A)** Serum T-AOC level. **(B)** Serum SOD level. **(C)** Serum MDA level. **(D)** Serum GSH-PX level. CON group (fed basal diet), LCHE (basal diet + 0.1% CHE), MCHE (basal diet + 0.2% CHE), HCHE (basal diet + 0.4% CHE).Mean ± SEM are shown (*n* = 8).Significant differences (*p* < 0.05) are denoted by the distinct letters a and b.

Figure 5 shows that on day 14 of the experiment, the T-AOC content in the serum of the MCHE group was significantly higher than that of the CON group (*p* < *0.05*), and the MDA content in the serum of the HCHE group was significantly lower than that of the CON group and the LCHE group (*p* < *0.05*). On day 28, the serum MDA level in the HCHE group was significantly lower than that in the CON group (*p* < *0.05*), and the serum GSH-PX level in the HCHE group was significantly higher than that in the CON group (*p* < *0.05*). On day 42, the serum SOD level in the HCHE group was significantly higher than that in the other groups (*p* < *0.05*), and the serum MDA level in the HCHE and MCHE groups was significantly lower than that in the CON group (*p* < *0.05*). In addition, the serum GSH-PX level was significantly higher in the HCHE group than in the empty CON group (*p* < *0.05*). On day 56, the serum SOD level was significantly higher in the HCHE group than in the CON group and the LCHE group (*p* < *0.05*), and the serum MDA level in the HCHE and MCHE groups was significantly lower than in the CON group (*p* < *0.05*). In addition, the serum GSH-PX level was significantly higher in the HCHE group than in the CON group and the LCHE group (*p* < *0.05*). Additionally, there was no significant difference between the HCHE and MCHE groups (*p* > *0.05*).

### 3.5 Analysis of gut microbiota

**Figure 6:**
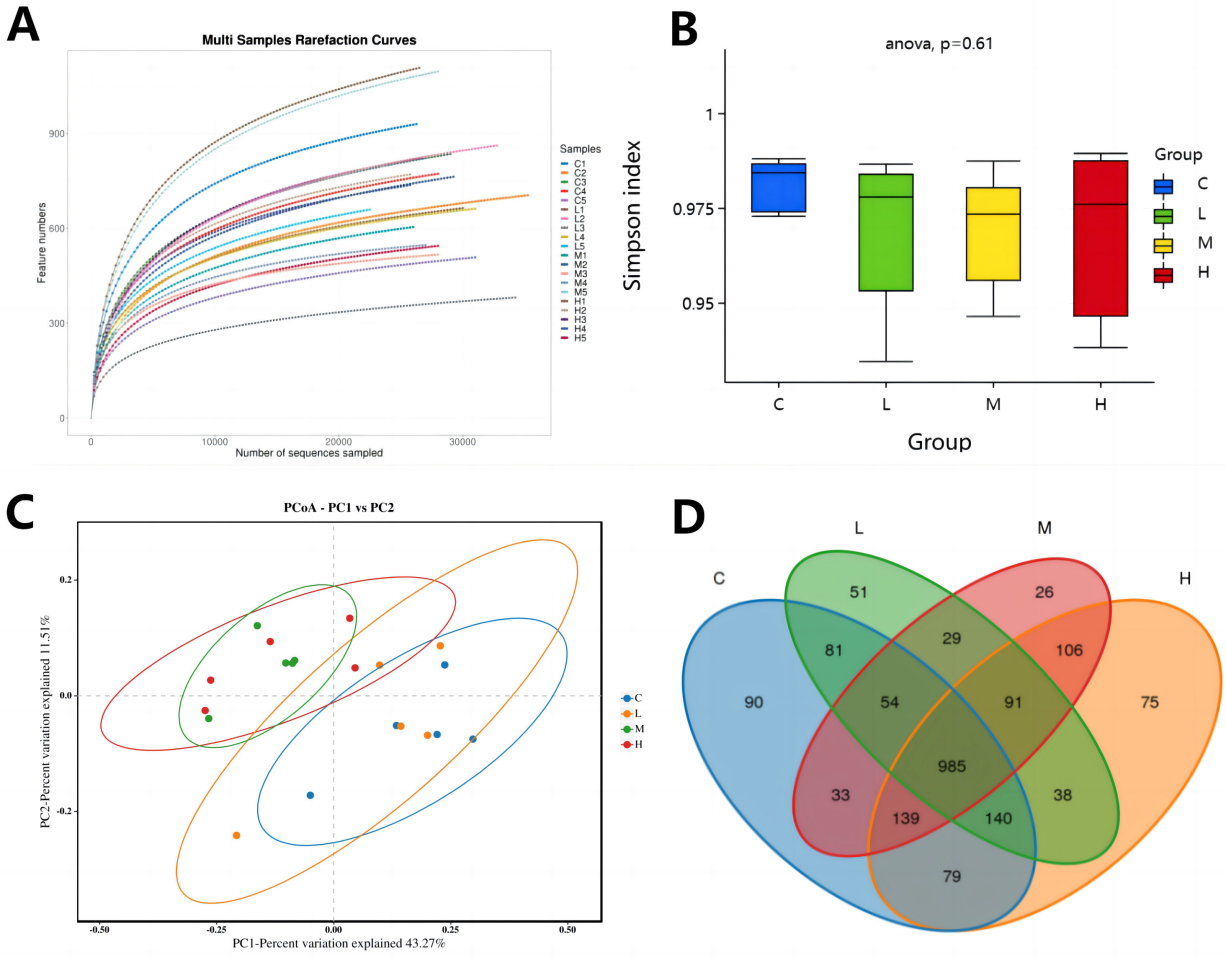
Effects of CHE on the fecal-like flora of calves **(A)** Sample dilution curve plot. **(B)** Grouped box plots of the AlPha diversity index. **(C)** Two-dimensional ordination plot of samples analyzed by Beta Diversity PCoA. **(D)** Venn diagram.C (fed basal diet). L (basal diet + 0.1% CHE), M (basal diet + 0.2% CHE), H (basal diet + 0.4% CHE). Mean ± SEM are shown (*n* = 5).

In this study, the effects of adding different doses of CHE to the diet on the gut microbiota of calves before and after weaning were investigated by 16S rRNA amplification and sequence analysis. Upon reaching 30,000 sequences, the rarefaction curve reached a plateau, indicating that sampling was sufficient and appropriate for this experiment (Figure 6A). Figure 6B shows the Simpson index of alpha diversity, which measures species diversity and is influenced by both species richness and community evenness. A higher Simpson's index value indicates greater species diversity in the sample. There were no significant differences in microbiota between groups (*p* > *0.05*). Figure 6C shows the beta diversity of the principal coordinate analysis (PCoA). There are differences in the composition of the diversity of the gut microbiota between the four calf groups. Principal component 1 (PC1) accounts for 43.27% of the total variance, while principal component 2 (PC2) accounts for 11.51%.

The LCHE group and the CON group are similar, while the HCHE group was different from the CON group and the MCHE group was more different from the CON group. The Venn diagram (Figure 6D) shows the number of common and unique OTUs in the samples. The CON group had a total of 1,511 OTUs, including 90 unique OTUs; the LCHE group had 1,418 OTUs, including 51 unique OTUs; the MCHE group had 1,437 OTUs, including 26 unique OTUs; and the HCHE group had 1,578 OTUs, including 75 unique OTUs.

**Figure 7:**
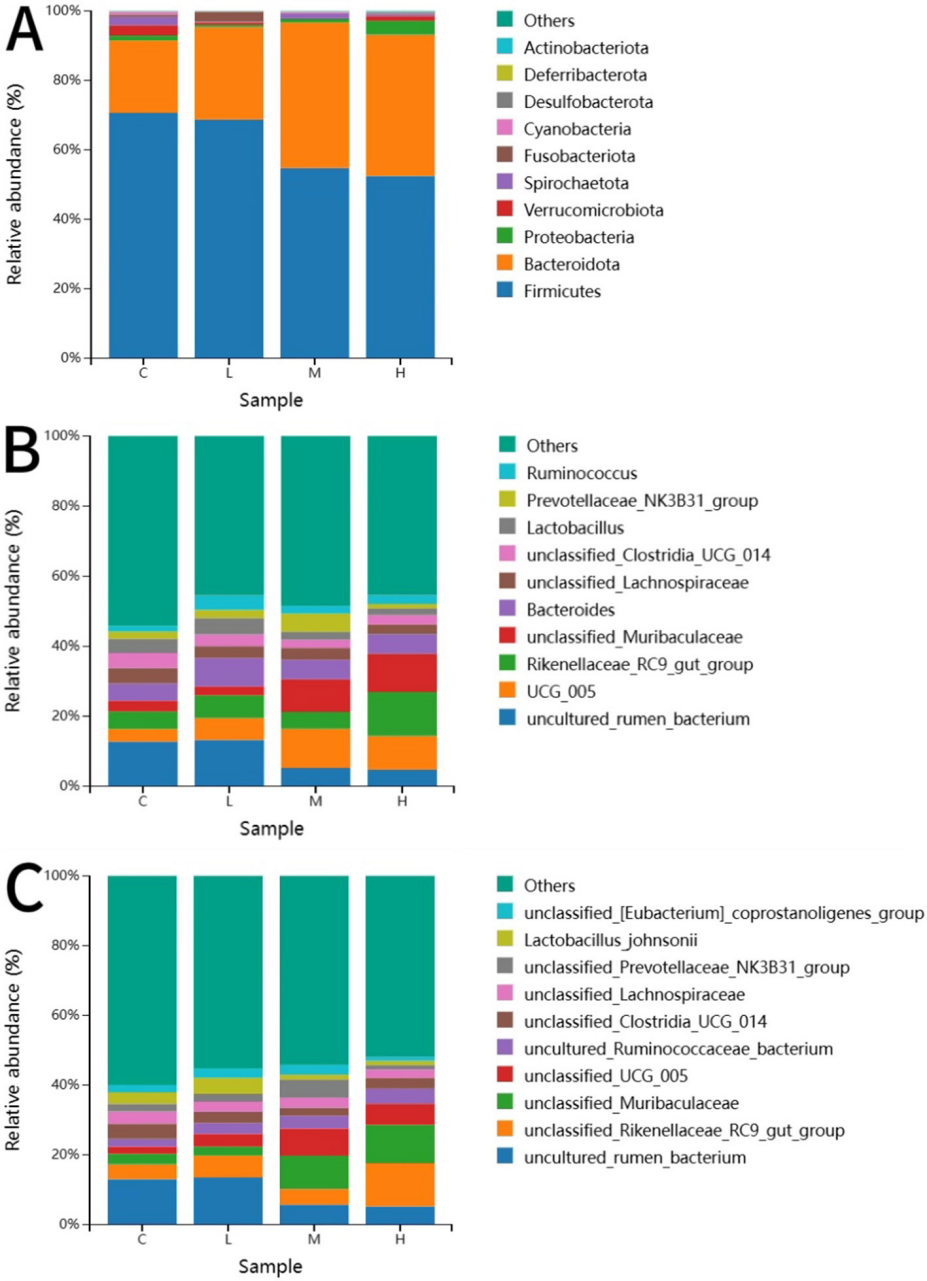
Effects of CHE on the fecal-like flora of calves. **(A)** Distribution of taxonomic composition at the Phyla level. **(B)** Distribution of taxonomic composition at the genus level. **(C)** Distribution of taxonomic composition at the species level. C (fed basal diet). L (basal diet + 0.1% CHE), M (basal diet + 0.2% CHE), H (basal diet + 0.4% CHE). Mean ± SEM are shown (*n* = 5).

Figure 7 shows the 10 most abundant gut microbiota at the phylum, genus and species level. At the phylum level, *Firmicutes* and *Bacteroidota* were the two predominant phyla in calf feces. The *Bacteroidota* content in the feces of the MCHE and HCHE groups was significantly higher than that of the CON group (*p* < *0.01*). At the genus level, the *unclassified Muribaculaceae* content in the MCHE and HCHE groups was significantly higher thanin the CON group and the LCHE group (*p* < *0.05*). The content of the genus *UCG-005* from the family *Ruminococcaceae* was higher in the LCHE, MCHE, and HCHE groups compared to the CON group, but the differences were not significant (*p* > *0.05*). The content of the genus *RC9* from the family *Lachnospiraceae* was higher in the HCHE group compared to the CON group, though the difference was not significant (*p* > *0.05*). At the species level, the *unclassified Muribaculaceae* content in the MCHE and HCHE groups was significantly higher than that in the CON group and the LCHE group (*p* < *0.05*). The *UCG-005 species* from the family *Ruminococcaceae* was significantly higher in the MCHE group compared to the CON group (*p* < *0.05*), while the LCHE and HCHE groups were higher than the CON group, but the differences were not significant (*p* > *0.05*).

### 3.6 Correlation analysis between gut microbiota abundance and growth performance

**Figure 8:**
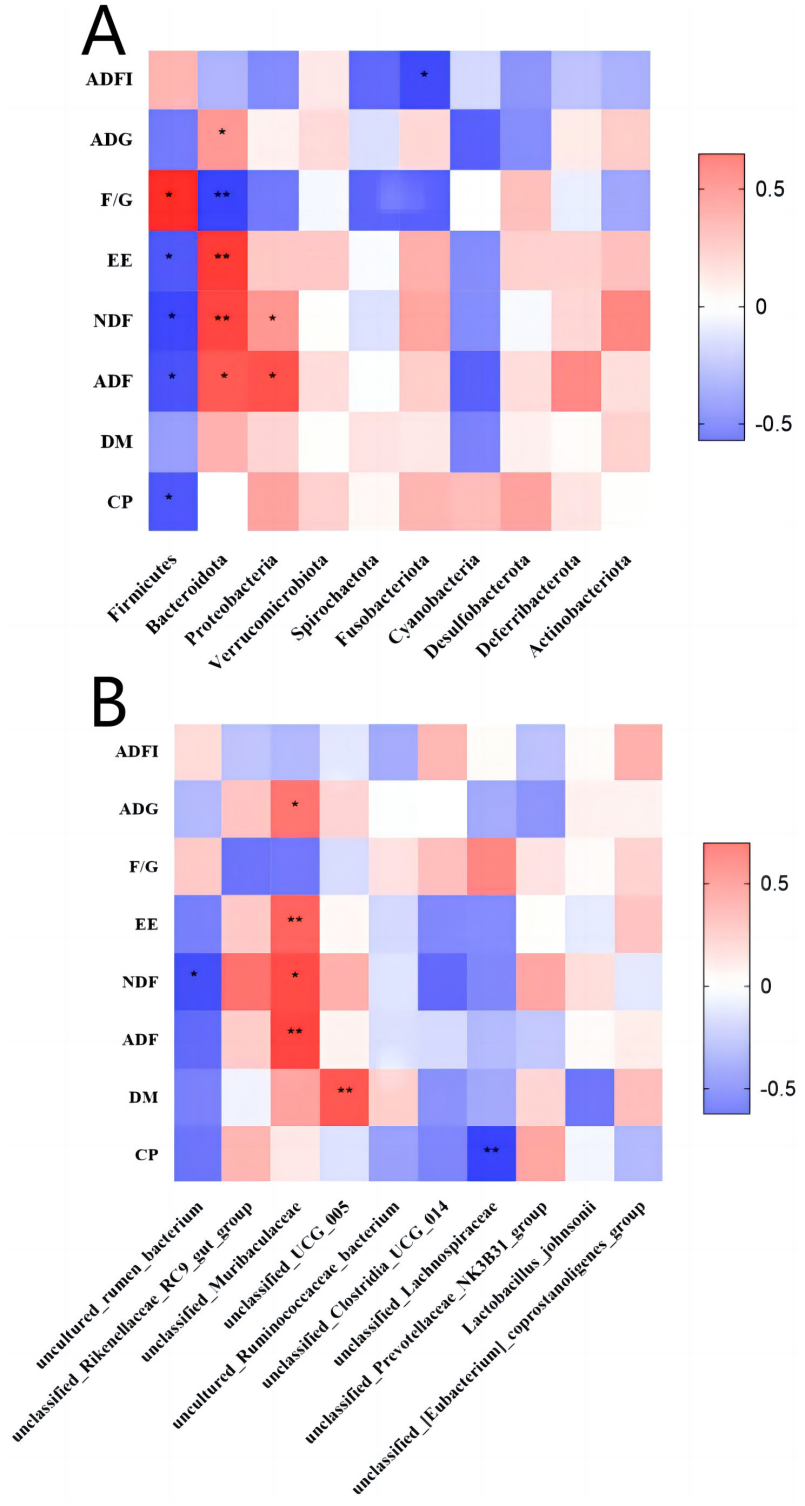
Correlation analysis chart. **(A)** Correlation analysis between phylum-level microbiota abundance and growth performance. **(B)** Correlation analysis between species-level microbiota abundance and growth performance. X-axis: microbial names. Y-axis: growth performance indicators. Red represents a positive correlation and blue represents a negative correlation. *p < *0.05*, **p < 0.01.

To further explore the impact of gut microbiota abundance on calf growth performance, we conducted a correlation analysis between microbiota abundance at the phylum and species levels and growth performance (Figure 8). As shown in Figure 8A, *Bacteroidota* is highly negatively correlated with F/G and highly positively correlated with EE and NDF. Figure 8B shows that *unclassified-Muribaculaceae* has a substantial positive correlation with EE and ADF, while *unclassified-UCG-005* shows a strong positive correlation with DM and *unclassified-Lachnospiraceae* has a strong negative correlation with CP.

## 4 Discussion

In this study, the CHE primarily contain several bioactive components, including chlorogenic acid, flavonoids, and polysaccharides, which exhibit significant antibacterial, anti-inflammatory, heat-clearing, detoxifying, and growth-promoting properties (27). So we investigated the potential of CHE as a feed additive in calf rearing, focusing on their effects on growth performance, serum immune indices and gut microbiota composition.

Growth performance, indicated by average daily gain and feed to gain ratio, reflects animal development and feed efficiency, respectively (28). Our results showed that there were no significant differences in dry matter intake between the CHE groups and the control group on days 0–56 and 43–56. However, the average daily gain was significantly higher in all CHE groups, with a correspondingly lower feed to gain ratio. The MCHE group had the best weight gain, while the HCHE group had the lowest feed to gain ratio, although these differences were not statistically significant. This indicated that the CHE additive improved calves' ADG, exhibited good palatability, and had no adverse effects on feed intake. Previous research suggests that the beneficial effects of herbal medicines are due to their polysaccharides, flavonoids and organic acids, which improve animal nutrition and growth. For example, the addition of traditional chinese herbal extract to calf diets can effectively improve growth performance this may be because the nutrients and some of the active ingredients of traditional Chinese medicine remaining in the residue promote the growth and development of animals, improve intestinal health, and thus promote the healthy growth of animals (29).

The apparent digestibility of nutrients reflects the ability of calves to digest and utilize nutrients from the diet and is an important indicator of feed quality (30, 31). In this trial, the apparent digestibility of EE, NDF and ADF was higher dose in the CHE groups than in the CON group, with the high dose and MCHE groups showing significant improvements. Among the dose groups, the MCHE group had the best digestibility of crude fat, while the HCHE group had the best digestibility of neutral and acid detergent fibers, although no significant differences were observed between the additive groups. The inclusion of plant residues in the diet has been shown to enhance nutrient digestion and absorption (32). Alkaloid compounds in herbal medicines can increase the activity of proteases and lipases, which facilitates the digestion and absorption of proteins and lipids (33). Therefore, it is likely that CHE improve the growth performance of calves by promoting nutrient digestibility and improving the gastrointestinal environment.

Growth hormone (GH) is an important indicator of growth promotion and metabolic regulation. GH, a peptide hormone secreted by pituitary cells, promotes tissue growth and increases protein and fat breakdown (34). IGF-1 in serum, a growth regulator, is closely associated with animal growth. It promotes cell proliferation, regulates protein synthesis and supports organ development and bone growth (35). The inclusion of herbal extracts in broiler diets has been shown to significantly increase serum GH and IGF-1 concentrations, leading to improved growth performance (36). Supplementation with chlorogenic acid at 1,000 mg/kg has been reported to enhance average daily gain and feed conversion efficiency in piglets, while also increasing serum albumin and IGF-1 levels (37). Additionally, flavonoids derived from herbal medicines have been demonstrated to positively modulate the hypothalamic-pituitary-adrenal axis, thereby enhancing GH and IGF-1 production and promoting growth performance in beef ewes (38). In this study, at day 28, serum GH and IGF-1 levels were higher in the CHE groups than in the CON group. Specifically, the MCHE group had the highest and significantly elevated GH levels, while the HCHE group had the highest and significantly elevated IGF-1 levels compared to the CON group. However, no significant differences were observed between the dose groups. It is therefore assumed that the flavonoids and chlorogenic acid components in CHE can increase the growth hormone level in calves and thus promote their growth and development.

Serum biochemical indicators are important indicators of nutrition, organ function and animal health (39). In this study, the effects of CHE feed additives on liver function (indicated by TP, ALT), kidney function (indicated by CREA, UREA), blood glucose level (GLU) and blood calcium level (CA) were investigated to determine the safe dosage of CHE. Numerous studies have evaluated the safety and efficacy of supplements based on biochemical indicators. For example, Previous studies have demonstrated that Artemisia leaves have no adverse effects on lamb health, as evidenced by serum levels of TP, ALT, and AST (40). Fathi et al. (41) found that administration of turmeric improved liver function of broiler chickens as shown by serum ALT and AST levels. In this study, serum TP and ALT levels in the CHE groups showed no significant differences compared to the CON group, indicating that the CHE does not impair protein synthesis in the liver or cause side effects on liver function. There were no significant differences in CREA and UREA levels between the groups, indicating that the CHE does not impair kidney function. Found in previous studies, chlorogenic acid in CHE could promote glucose uptake by up-regulating the expression of glucose transporter 2 (GLUT2) and phosphofructokinase (PFK), which activates the thermogenesis of brown adipose tissue and promotes the endocrine release of fibroblast growth factor-2 (42). It has been found that the addition of different concentrations of CHE to the diet of laying hens resulted in a higher trend in serum GLU levels with increasing herbal concentration (43). On day 56 of this trial, serum GLU levels in the CHE groups were within the normal range and significantly higher than in the CON group (44), with the low and high groups showing significant increases. The MCHE group had the highest value, although there were no significant differences between the CHE groups. This suggests that GLU levels may indirectly reflect weight gain in calves and that chlorogenic acid in CHE may improve weight gain while maintaining normal serum GLU levels. CA is an important component of teeth and bone, contributes to the transmission of neuromuscular excitability, regulates muscle contraction, and is essential for enzyme-catalyzed chemical reactions (45). At day 28, serum CA levels were within the normal range in all CHE groups and higher than in the CON group, with significantly higher levels in the medium and HCHE groups. Among the doses, the medium and high doses had the highest CA levels, with no significant differences between them. This indicates that the CHE can improve the growth performance of calves by increasing the CA content. Therefore, the MCHE and HCHE can improve serum GLU and CA levels without affecting liver or kidney functions.

Serum concentrations of IgA and IgG are important indicators of immune function, which play important roles in antibacterial and antiviral responses (46). IgA acts as an inhibitor of inflammatory responses, preventing pathogen adhesion to mucosal surfaces and thereby resisting mucosal infections (47). IgG, which accounts for ~75% of the total immunoglobulins, activates complement system and neutralizes toxins, reflecting the overall immune status of the body (48). Many traditional Chinese herbs have immuno-modulatory effects, stimulating the immune system, activating immune cells, and enhancing the synthesis of antibodies, thus improving immune function and promoting animal health (49). Numerous studies have demonstrated that *Astragalus* polysaccharides (APS) in *Astragalus* has significant immunomodulatory effects both *in vivo* and *vitro* in immunosuppression animal models and it also enhances the immune effects of normal animals (50). Dietary supplementation with 0.3% *Astragalus* significantly elevated serum IgA and IgM levels in lambs, indicating *Astragalus* improves immune function in early weaned lambs (51). Similarly, the inclusion of *Honeysuckle* extract in geese diets was shown to significantly increase serum IgA and IgG levels (52). In this study, although there were no significant differences in serum IgA levels between groups, the CHE groups had higher levels compared to the CON group, suggesting that the CHE promoted IgA expression. At day 56, serum IgG levels were higher in all CHE groups than in the CON group, with significant increases in the medium and HCHE groups. There were no significant differences between doses. IL-8 is a pro-inflammatory cytokine secreted by monocytes, macrophages, and endothelial cells and it recruits neutrophils to inflammatory sites, activates inflammatory cells and induces cell proliferation and the release of inflammatory substances (53). IFN-γ is a highly biologically active non-specific antiviral substance with immunoregulatory and differentiation-inducing functions, playing a key role in cell-mediated immune responses (54). The inclusion of a CHE compound in laying hen feed was shown to increase serum IFN-γ levels while reducing pro-inflammatory cytokine IL-8 levels, indicating that Chinese herbal compounds may enhance immune function and suppress inflammatory responses (8). This effect is presumed to be due to the stimulation of small intestine lymphocyte proliferation by the herbs. In this study, there were no significant differences in IL-8 levels among the groups, indicating similar levels across groups. On day 28, serum IFN-γ levels were higher in all CHE groups compared to the CON group, with the HCHE group showing the highest level, significantly higher than the CON group. There were no significant differences among the additive doses, but IFN-γ levels increased linearly with the dose. This may be due to chlorogenic acid in CHE can directly act on the NF-kB signaling pathway, regulating the expression of anti-inflammatory and pro-inflammatory factors. In addition, chlorogenic acid in CHE prevents the damage caused by inflammation by regulating the expression of related proteins and genes in the inflammatory response (55). In conclusion, CHE can promote the expression of IgG and IFN-γ in calves, with the 2% and 4% doses showing the most effective results.

The antioxidant capacity of dairy calves during the lactation period is closely related to their overall health. Increased antioxidant capacity strengthens the body's ability to resist oxidative damage (56, 57). The antioxidant capacity of animals can be determined by changes in serum GSH-PX and SOD activity and MDA levels. MDA, the end product of lipid peroxidation, can cause cell damage. In contrast, GSH-PX and SOD can neutralize free radicals and inhibit MDA formation, thereby maintaining the oxidative balance in the body (58–60). Numerous studies have shown that traditional chinese herbal feed additives can improve the antioxidant capacity of animals, Because chlorogenic acid in chinese herbal feed has a comprehensive antioxidant function. The molecular structure of chlorogenic acid contains five active hydroxyl groups and one carboxyl group, which gives chlorogenic acid its natural antioxidant properties (61). For example, *Honeysuckle* extract has been shown to enhance antioxidant capacity by increasing ferric reducing antioxidant power (FRAP) and 2,2′-azino-bis(3-ethylbenzothiazoline-6-sulfonic acid) (ABTS) radical scavenging activity (62). Dietary administration of APS was found to improve antioxidant capacity in broilers by elevating serum activities of SOD and GSH-PX, while reducing MDA concentrations (63). Similarly, supplementation with 10% *Astragalus* residues in the diet of fattening pigs significantly increased their antioxidant capacity (64). Furthermore, magnolol supplementation at varying doses enhanced antioxidant capacity in broilers, as evidenced by increased T-SOD activity in serum and intestinal mucosa, along with reduced MDA levels (65). In this study, serum T-AOC levels at day 14 were higher in the CHE groups than in the CON group, with the MCHE group having the highest and significantly higher levels than the CON group. Serum SOD levels on days 42 and 56 were higher in the CHE groups than in the CON group, with the HCHE group having the highest and significantly higher levels than the CON group. Serum MDA levels on days 14, 28, 42, and 56 were lower in the CHE groups compared to the CON group, with the MCHE and HCHE groups having the lowest and significantly lower levels than the CON group. Serum GSH-PX levels were higher in the CHE groups than in the CON group on days 28, 42, and 56, with the HCHE group having the highest and significantly higher levels than the CON group. These results are consistent with previous studies and suggest that CHE at different doses can improve the antioxidant capacity of dairy calves, with MCHE and HCHE groups doses showing better effects.

The gut microbiota plays a crucial role in the physiological processes of nutrient absorption, growth and development, and immune regulation in dairy calves (66, 67). The diversity and richness of microbial communities can lead to differences in digestive capacity (68, 69), which in turn affects growth performance. Studies have shown that chlorogenic acid, flavonoids, phenylpropanoids, alkaloids and terpenes in herbal supplements can inhibit the growth of harmful bacteria and prevent the formation of bacterial biofilms, thereby regulating gut health and improving growth performance (70). Regarding microbial diversity, although the addition of CHE to the diet had no significant effect on the Simpson index, the β-diversity results showed that the microbial community structure in the MCHE and HCHE groups differed from that of the CON group. Greater microbial diversity is associated with improved production performance, a robust gut microbiome and increased resistance to invasive species (71). Regarding the composition of the gut microbiota, the proportion of *Bacteroidota* in the feces of the MCHE and HCHE groups was significantly higher compared to the CON group. Previous studies have identified *Firmicutes* and *Bacteroidetes* as the two dominant phyla. Members of the *Bacteroidetes* are primarily responsible for protein hydrolysis and carbohydrate degradation (29). At the genus and species level, the abundance of the genus *Muribaculaceae* and *Muribaculaceae* species was significantly higher in the MCHE and HCHE groups than in the CON group. *Muribaculaceae* is a common gut symbiont in animals that is crucial for host health by maintaining intestinal homeostasis through competitive relationships, production of antimicrobial substances and modulation of the immune system (72). Chlorogenic acid in chinese herbal feed can increase the relative abundance of *Bacteroidetes* and *Firmicutes* in the intestine, and reduce the relative abundance of *Proteobacteria*, and increase the diversity of intestinal microorganisms, indicating that the chlorogenic acid of CHE feed played an important role in regulating intestinal organisms in this study (73). In addition, *Muribaculaceae* bacteria contribute to the production of beneficial short-chain fatty acids by breaking down complex polysaccharides in the diet, which plays an important role in gut function and metabolism. At the species level, the relative abundance of *Ruminococcaceae-UCG-005* was significantly higher in the MCHE group compared to the CON group. The relative abundance of *Ruminococcaceae* in the bovine rumen is positively correlated with digestibility (74). *Ruminococcaceae-UCG-005* is involved in the breakdown of cellulose and the digestion of starch, converts complex polysaccharides into various nutrients required by the host and plays a crucial role in the metabolic functions of dairy calves (75). In summary, the results show that the MCHE and HCHE groups have better intestinal stability compared to the CON and LCHE groups. ncreasing the number of harmful microorganisms in the gut helps maintain normal physiological functions, thereby improving gastrointestinal digestion and increasing the growth performance of dairy calves (76).

Therefore, this study investigated the correlation between gut microbiota abundance and growth performance indicators in dairy calves to explore the effects of CHE on growth performance by modulating the abundance of the gut microbiota. Found that *Bacteroidota* was strongly negatively correlated with F/G and strongly positively correlated with EE and NDF. *Unclassified-Muribaculaceae* showed a highly significant positive correlation with EE and ADF, while *Ruminococcaceae-UCG-005* showed a highly positive correlation with DM. It has been demonstrated that *Ruminococcaceae* abundance exhibits a strong positive correlation with body weight and ADG (77). Additionally, a significant positive correlation was observed between the abundance of *norank_f_Bacteroidales_UCG-001* and ADF digestibility (74). Furthermore, the relative abundance of *Ruminococcaceae* was positively associated with feed efficiency and feed intake. Improved CF utilization in dairy calves, which is closely linked to higher DM digestibility.These findings suggest that *Ruminococcaceae* abundance plays a significant role in promoting nutrient utilization and growth performance (78). Thus, CHE intake may improve growth performance by increasing the number of beneficial gut bacteria, which in turn promotes more effective nutrient digestion. We also found that *unclassified-Lachnospiraceae* were strongly negatively correlated with CP. The relationship between *Lachnospiraceae* and CP is currently limited and further studies are needed to investigate the underlying mechanisms.

## 5 Conclusion

In conclusion, the addition of CHE to the feed significantly improves the average daily weight gain of Holstein calves, reduces the feed-to-gain ratio and improves the structure of the gut microbiota, thereby increasing growth performance. In addition, CHE can also enhance immune function and antioxidant function. Given the observed improvements in growth performance and health benefits, a clinical dosage of 0.2% CHE supplement is recommended for optimal results.

## Data Availability

The datasets presented in this study can be found in online repositories. The names of the repository/repositories and accession number(s) can be found in the article/supplementary material.
